# Psychological and clinical-epidemiological profile of poisoning in Nepal: an institutional experience

**DOI:** 10.12688/f1000research.54327.2

**Published:** 2021-08-31

**Authors:** Angela Basnet, Dhan Shrestha, Sabin Chaulagain, Ashok Thapa, Manoj Khadka, Bishal Regmi, Manita Khadka, Kabita Adhikari, Anil Jung Thapa, Sakar Pokharel, Kaushal Kumar Singh, Prajwal Syangtang, Surakchha Adhikari

**Affiliations:** 1 Department of Internal Medicine, Scheer Memorial Adventist Hospital, Banepa, Nepal; 2 Department of Emergency Medicine, Mangalbare Hospital, Urlabari, Morang, Nepal; 3 Nepalese Army Institute of Health Sciences (NAIHS), Kathmandu, Nepal

**Keywords:** organophosphates; Nepal; suicide

## Abstract

Background

Poisoning has become a major public health problem, with the intent in most cases being self-harm and commit suicide. This study highlights the psychological and clinical-epidemiological profile of patients visiting Scheer Memorial Adventist Hospital after poisoning.

Methods

This retrospective record-based study was done among poisoning patients of a hospital in Nepal from 1st January 2018 to 31st December 2020. Data were analyzed using STATA version-15.

Results

Out of 134 total poisoning cases, 71 had consumed organophosphate compounds. The majority of the cases were female (59.2% in organophosphate groups, 69.8% in non-organophosphate groups). The circumstances of poisoning were mostly suicidal (95.8% in organophosphate groups, 90.5% in non-organophosphate groups) and the reasons for this being mostly family disputes. Organophosphate groups had 8.41 times higher odds of having complications when compared to non-organophosphorus compounds.

Conclusions

The majority of the poisoning cases were suicidal in nature and family disputes being the major reason for the intake of a poisonous substance. This demands that more attention be given to psychological and family counseling to resolve any disputes, as well as psychological management of poisoning cases after medical management. Also, a strong regulatory mechanism should be imposed to control the easy access to poisonous substances.

## Introduction

As per reports from the World Health Organization (WHO), about 0.3 million people die each year due to various acute poisonings. ^1^ The substances most commonly used in poisoning are agricultural pesticides (mostly organophosphates) in developing countries and misuse of drugs in developed countries. The common reason for most of the poisoning cases is the intention of self-harm. ^1^ WHO estimates that pesticide self-poisoning accounts for around 20% of global suicides. ^2^ Around 60% of the estimated annual deaths from self-harm in rural Asia are due to pesticide poisoning, and organophosphates account for around two-thirds of these deaths. ^3^ In low and middle-income countries (LMICs) like Nepal, where 66% population is directly engaged in agriculture, pesticides have become the preferred means of suicide due to agricultural intensification and their easy availability. ^4^

Limited information is available regarding the pesticide poisoning scenario in Nepal as the national routine Health Management and Information System (HMIS) doesn’t include it. ^5^ Also, the psychological aspect associated with poisoning in Nepal is not addressed much in the literature although the medical aspect is given high priority.

Our study aims to address the psychological issues of poisoning with the main focus on its intent and its motives. Apart from this, the study also highlights the clinical and epidemiological profile among patients presenting to the emergency department of a hospital in Nepal following intentional or accidental poisoning.

## Methods

### Study design and setting

This is a retrospective hospital record-based study done among patients presenting to the emergency department of Scheer Memorial Adventist Hospital (SMAH) following confirmed or suspected poisoning.

### Study sample

All the patients presenting to the emergency department of SMAH following confirmed or suspected poisoning from 1 ^st^ January 2018 to 31 ^st^ December 2020 were included in the study. The study was conducted from 28 ^th^ February to 14 ^th^ March 2021. Patients were either admitted to the ICU or General ward, referred to other centers, or were brought in already deceased. Patients who had not been exposed to poison were excluded from the study.

### Study instruments

Different sets of data extraction sheets were prepared for both general compound poisoning and organophosphorus compound poisoning respectively. Each questionnaire consists of demographic variables like age, sex, marital status, religion, profession, external variables like reasons for taking poisons, manner of taking poisons, history of suicide attempts, history of psychiatric illness (as reported by the patient), and clinical variables like presenting signs and symptoms, along with outcome and complications. The study instrument is available in **Extended data**. Data were recorded at the clinical encounter through a combination of the physician, nurse, and social work documentation in hospital records. The records regarding the poisoning and suicidal cases can be found in the medical record section of the hospital. Also, a special stamp was applied to the files for easy retrieval of the file records when needed.

### Statistical methods

The data were extracted to Microsoft Excel-13, then imported and analyzed using STATA-15 (Stata, RRID:SCR_012763). Descriptive variables were presented in simple frequency tables. Continuous variables were expressed in terms of mean, standard deviation. Binary logistic regression analysis was performed for the dependent outcome of ‘complications’ with the occurrence of complications as ‘1’ and no complications as ‘0’ among organophosphorus and non-organophosphorus compounds. Subsequently, further independent variables studied were explored to get adjusted odds complications within the organophosphorus compound group.

### Ethical consideration

The study was approved by the Institutional Review Committee of SMAH (Ref no: 2608). The data were collected from the hospital records and as a part of the standard workup and treatment of the patients. The ethics committee waived the requirement for patient consent due to the retrospective and anonymous nature of the data.

## Results

Out of 159 total poisoning cases, 71 had consumed organophosphate compounds while 63 had consumed non-organophosphate compounds like non-organophosphate pesticides, drugs, and rodenticides. Since Aluminum phosphide (n = 25) cases had unique findings (all patients being suicidal in intent and with a high case fatality rate of 84%) compared to other non-organophosphate poisoning cases, aluminum phosphide cases have been reported elsewhere as a case series. Hence they were not included in the analysis of the non-organophosphate group.

### General characteristics

The mean age of study participants in the organophosphate (OP) group was 31.72 (±14.07) years, while that in the non-organophosphate (Non-OP) group was 27.41 (±9.79) years. More than half of the cases in both groups were female (59.2% in OP, 69.8% in Non-OP). The majority of the study participants were married (80.3% in OP and 47.6% in Non-OP). Hinduism was the major religion in both groups (56.3% in OP and 44.4% in Non-OP). The intent of intake of substance was mostly suicidal (95.8% in OP and 90.5% in Non-OP) although the majority of them had no prior suicide attempt (91.5% in OP and 96.8% in Non-OP) and had no psychiatric history (91.5% in OP and 95.2% in Non-OP). The reason for intake being mostly a dispute (63.4 % in OP group). More than half of the OP patients (56.3%) and most of the Non-OP group patients (95.2%) brought to the hospital were conscious. Emesis was induced at home in most of the patients of both groups (59.2% in OP and 41.3% in Non-OP). Likewise, most of the study participants had gastric lavage (85.9% in OP and 47.6% in Non-OP) and charcoal lavage (81.7% in OP and 20.6% in Non-OP) done in the emergency block of the hospital ( Table 1).

**Table 1.** Baseline patient details among OP group and Non-OP group.

Variables		OP (n = 71) n (%)	Non-OP (n = 63) n (%)
Age in years	Mean ± SD (IQR)	31.72 ± 14.068	27.413 ± 9.7941
	Missing	0 (0)	2 (3.2)
Gender	Female	42 (59.2)	44 (69.8)
	Male	29 (40.8)	19 (30.2)
Marital status	Married	57 (80.3)	30 (47.6)
	Unmarried	12 (16.9)	15 (23.8)
	Undisclosed	2 (2.8)	16 (25.4)
	Divorce	0 (0)	1 (1.6)
	Widow	0 (0)	1 (1.6)
Religion	Hindu	40 (56.3)	28 (44.4)
	Undisclosed	25 (35.2)	32 (50.8)
	Buddhist	6 (8.5)	3 (4.8)
Intent of intake of substance	Suicidal	68 (95.8)	57 (90.5)
	Accidental	3 (4.2)	5 (7.9)
	Not disclosed	0 (0)	1 (1.6)
Reason for intake of a substance	Dispute	45 (63.4)	
	Not disclosed	25 (35.2)	
	Depression	1 (1.4)	
Prior suicide attempt	No	65 (91.5)	61 (96.8)
	Yes	6 (8.5)	2 (3.2)
Psychiatric disorder	No	65 (91.5)	60 (95.2)
	Yes	6 (8.5)	2 (3.2)
	Doubtful	0 (0)	1 (1.6)
Cigarette smoking	No	53 (74.6)	56 (88.9)
	Yes	18 (25.4)	7 (11.1)
Drinking alcohol	No	42 (59.2)	46 (73.0)
	Yes	29 (40.8)	17 (27.0)
Conscious level	Conscious	40 (56.3)	60 (95.2)
	Semiconscious	24 (33.8)	2 (3.2)
	Unconscious	7 (9.9)	1 (1.6)
Emesis at home	Yes	42 (59.2)	26 (41.3)
	No	29 (40.8)	37 (58.7)
Gastric lavage	Done	61 (85.9)	30 (47.6)
	Not done	10 (14.1)	33 (52.4)
Charcoal lavage	Done	58 (81.7)	13 (20.6)
	Not done	13 (18.3)	50 (79.4)
Atropinization in other centers	No	58 (81.7)	
	Yes	9 (12.7)	
	Missing	4 (5.6%)	

### Baseline vitals and outcome of patients

More than half of the patients in the OP group had a normal pulse (n = 41, 57.7%), while 38 % had tachycardia. Likewise, more than three-fourths of the patients in the Non-OP group (79.4%) had a normal pulse. Among OP patients, the mean systolic blood pressure (SBP) was 124.93 (±22.66) mm Hg, and the mean diastolic blood pressure (DBP) was 79.13 (±13.47) mm Hg. Similarly in Non-OP patients, the mean SBP was 116.03 (±14.54) mm Hg and the mean DBP was 75.87 (±10.57) mm Hg. The majority of the patients had temperatures within the normal range (93.0% in OP and 95.2% in Non-OP). Most patients with OP poisoning (n = 43, 60.6%) had increased respiratory rate while Non-OP group patients (n = 40, 63.5%) had normal respiratory rate. The mean POP (Peradeniya Organophosphorus Poisoning) score for OP patients was 2.20 (±1.685), with more than three-fourths patients having mild scores (77.5%) and only 1.4% being severe. Almost all patients (97.2% in OP and 95.2% in Non-OP) were admitted to the ICU of the hospital. All patients with Non-OP poisoning improved and were sent home whereas 5.6 % of OP patients were dead either during treatment or brought dead to the hospital ( Table 2).

**Table 2.** Vitals at presentation and outcome of patients of OP-group versus Non-OP-group.

Variables		OP n (%)	Non-OP n (%)
Pulse	<60	0 (0)	2 (3.2)
	60-100	41 (57.7)	50 (79.4)
	>100	27 (38.0)	11 (17.5)
	Missing	3 (4.2)	0 (0)
SBP	Mean ± SD	124.93 ± 22.66	116.03 ± 14.54
	Missing	2 (2.8)	0 (0)
DBP	Mean ± SD	79.13 ± 13.47	75.87 ± 10.57
	Missing	2 (2.8)	0 (0)
Temp	<97	3 (4.2)	3 (4.8)
	97-100.4	66 (93.0)	60 (95.2)
	Missing	2 (2.8)	0 (0)
RR (Respiratory rate)	15-20	26 (36.6)	40 (63.5)
	>20	43 (60.6)	23 (36.5)
	Missing	2 (2.8)	0 (0)
POP scale category	Mild (0-3)	55 (77.5)	
	Moderate (4-7)	13 (18.3)	
	Severe (8-11)	1 (1.4)	
	Missing	2 (2.8)	
	Mean ± SD	2.20 ± 1.68	
Complication	No	40 (56.3)	58 (92.1)
	Yes	29 (40.8)	5 (7.9)
	Unknown	2 (2.8)	0 (0)
Admission in hospital	ICU admission	69(97.2)	60(95.2)
	Referred to other centers	1(1.4)	0(0)
	Death on arrival	1(1.4)	0(0)
	General ward	0(0)	3(4.8)
Hospital stay	Mean ± SD	9.95 ± 4.21	
	Missing	5(7)	
Final outcome	Improved & sent home	64(90.1)	63(100.0)
	Deceased during treatment	3(4.2)	0(0)
	Missing	2(2.8)	0(0)
	Death on arrival	1(1.4)	0(0)
	Leave against medical advice (LAMA)	1(1.4)	0(0)

### Occupation of OP and Non-OP groups

The majority of the participants had their occupation not disclosed (57.7% in OP, 60.3% in Non-OP). Most of the remaining individuals in both OP and Non-OP groups were either house managers (18.3% in OP, 6.3% in Non-OP), students (11.3% in OP, 17.5% in Non-OP), or farmers (5.6% in OP, 6.3% in Non-OP) ( **Extended data** **Table 1 and 2**).

### Reason for ingestion of the substance

A family dispute was the most common reason for taking the substance among both OP groups (46.5%) and Non-OP groups (28.6%) ( **Extended data** **Table 1 and 2**).

### Substance ingested in the OP and Non-OP groups

The common OP substances ingested were a combination of Chlorpyrifos and Cypermethrin (28.2 %), Chlorpyrifos (19.7%), Dichlorvos (18.3%), Methyl parathion (7.0%), a combination of Triazophos and Deltamethrin (2.8%), and Malathion (1.4%). Around a quarter of the individuals (22.5%) had the OP substance they ingested not specified ( **Extended data** **Table 3**).

**Table 3.** Binary Logistic Regression for dependent outcome complication (Note: ® = Reference category).

Complication cat	Odds Ratio	Std. Err.	Z	P>|z|	[95% Conf. Interval]	
Non-op®						
OP	8.41	4.424045	4.05	0.000	2.999335	23.58126
Constant	.0862069	.0401803	−5.26	0.000	.0345783	.2149215

Among Non-OP groups, almost half of the participants ingested either non-organophosphate insecticides (25.4%) or drugs (22.2%). The most commonly ingested non-organophosphate insecticides were Cypermethrin (n = 4, 25%) and Emamectin benzoate (n = 3, 18.8%), whereas the most commonly ingested drug was NSAIDS (n = 11, 78.6 %), either along with other drugs or alone. Among NSAIDs, Paracetamol was the most commonly ingested drug (n = 9, 81.8%) ( **Extended data** **Table 2 and 3**).

### Different symptoms among OP ingested individuals

More than two-thirds of the participants presented with vomiting (n = 49, 69.0%), more than half with miosis (n = 42, 59.2%) and almost half with nausea (n = 34, 47.9%).

The most common nicotinic presentation was hypertension (n = 14, 19.7%) and tachycardia (n = 14, 19.7%), whereas the most common central presentation was confusion (n = 24, 33.8%) and anxiety (n = 18, 25.4%) ( **Extended data Table 4**).

### Complications encountered

More than half of the participants (n = 40, 56.3%) in the OP group and 92.1 % participants in the Non-OP didn’t have complications. Among the participants who experienced complications, the most commonly encountered among OP groups was aspiration pneumonia (n = 12, 38.7%), either alone or in combination with other complications, and among the Non-OP group the most commonly encountered was bradycardia (n = 3, 60%) ( **Extended data Table 5 and 6**).

### Binary Logistic Regression for dependent outcome complication

Binary Logistic Regression for dependent outcome complication (Yes = 1, No = 0) taking other poison categories as independent variables showed OP category has 8.41 times higher odds of having complications (95% CI: 2.999-23.581, P < 0.001) ( Table 3).

### Binary Logistic Regression for dependent outcome complication taking other independent variables among OP -group

Different independent variables couldn’t show association of developing a complication among the OP poisoning group with good precision ( **Extended data Table 7**).

## Discussions

Acute Pesticide Poisoning is a major health-care problem and a frequent medical emergency in developing countries with high case fatality. ^1, 5, 6^ This retrospective hospital-based study intends to explore various factors contributing to the exposure of poison and the outcome. Recent national, as well as international studies, suggest that younger people have emerged as a high-risk age group. ^1, 5– 7^ Our study also showed that the mean age in years in OP poisoning cases was 31.72 (±14.068). The fact that poisoning is more common among young adults reflects their vulnerability to stress, maladjustment, and immature psychological coping mechanisms at a time of life’s major stressors. ^8, 9^

In our study, females were more involved in organophosphate poisoning as well as in other acute poisonings which was consistent with other studies conducted in different national hospitals. ^5, 10– 12^ In contrary to this finding, a study done at Liaquat University of Medical and Health Sciences, Pakistan over one year period showed only 22% were females. ^13^ More vulnerability of females to suicide attempts than males in developing countries like Nepal points towards various social and cultural factors revealing inequality. Factors like early marriages, fewer opportunities for education, domestic violence, abusive spouses, problematic love and marital relationships, and unwanted pregnancies, contribute to more suicide attempts by women. ^7, 9, 14, 15^ According to the Nepal Maternal Mortality and Morbidity (MMM) study 2008/09, the leading cause of death among women of reproductive age was suicide, comprising 16 percent of all deaths increasing from 10.2 percent in 1998.

This study revealed that more than 90 percent of the patients intentionally consumed the poison (self-poisoning) as a suicidal attempt. A similar study was done in Bir Hospital, Nepal which showed 97% intentional poisoning. ^12^ Also, a study done in Turkey revealed that deliberate self-poisoning was the most common cause of poisoning (58.6%), followed by accidental exposure (39.1%). ^16^ A study from Nepal that examined attempted suicide showed that the commonest mode of suicide among all attempted cases was OP poisoning. ^17^ Pesticides becoming the first choice of method of suicide in rural agricultural communities is attributed to easy availability and no restrictions on buying and selling. ^5, 14, 15, 18^ To support this, we can look at the fact that in Sri Lanka, the suicide rates halved in the mid 1990s after a series of legislative activities systematically banned the most highly toxic pesticides. ^19^ The ease of availability of lethal means of self-harm may influence patterns of suicide. Suicidal impulses are often short-lived and if we can buy some time by making the means of suicide less readily available, a proportion of suicides will be prevented. ^18, 20, 21^

Most of the suicidal cases were impulsive in nature and the main triggering factor for attempting suicide was found to be interpersonal conflict (family disputes) in our study. In many other studies, interpersonal conflicts involving domestic problems are the main precipitating factors. ^22– 24^ However, various studies done in India, Sri Lanka, and other countries showed that psychiatric illnesses like depression are associated with high-risk attempts. ^25– 27^ It was not significant in our study due to inadequate data and incomplete history provided by the patient’s party. Since we relied completely on the history given by patients for psychiatric illness, its prevalence among them cannot be verified and can be more than what is reported. Evidence also suggests that there is a lesser prevalence of psychiatric disorders in individuals involved in suicidal behavior in LMICs. It may reflect the suicide methods used in LMICs (e.g., pesticide ingestion) which are mostly impulsive acts with low suicidal intent. ^28^

As is commonly observed in different studies, organophosphorus compounds are common mode of poisoning in our study followed by drugs and rodenticides. ^5, 10, 29^ However, some studies showed a higher percentage of rodenticides compared to drugs. ^11, 30^ Among different organophosphorus compounds, our study showed the combination of chlorpyrifos and cypermethrin to be the most common. Previous studies from Nepal and other countries report methyl parathion and dichlorvos as the commonest causes of OP poisoning. ^11^ But contrary to these studies and as mentioned by the National Forensic Science Laboratory, ^29^ the use of a combination of chlorpyrifos and cypermethrin seems to be increasing. This might be because compounds like methyl parathion and dichlorvos, previously very common compounds of poisoning, have been banned from the country.

In our study 77.5 percent of patients fall under mild severity as per POP score. It has been shown by Senanayeke *et al*. that the severity, morbidity, and mortality of OP poisoned patients can be predicted through the POP score. ^31^ Perhaps we can conclude the pertinent factor for improved survival in our study was the mild severity status of the patients at presentation. A previous study from Karachi also showed a similar relation between POP score and severity. ^32^

In this study, 26.86 per cent of all poisoning cases developed complications, very similar to one of the studies from Central Nepal. ^10^ This shows that the majority of the patients recovered completely without any complications and sequelae which might be because of various reasons like adequate and proper management, ingestion of low doses, and induction of vomiting at home as seen in this study as well. Aspiration pneumonia was the commonest complication in the OP group with bradycardia being common in the Non-OP group. Other studies are also found in favor of these findings. ^10, 33^ The intermediate syndrome was also found in a good percentage (5 per cent) among the OP group similar to the findings of a study from eastern Nepal ^3^ but significantly low compared to a study from India (29.4 percent). ^34^

90 per cent of patients with OP poisoning were recovered while 100 percent of patients recovered with other Non-OP poisoning. The case fatality was 4.4 percent among OP poisoning patients excluding the cases whose outcome couldn’t be evaluated such as referred cases and LAMA. The fatality rate was lower as compared to various studies done in Nepal and other South Asian countries. ^10, 35, 36^

The study being retrospective and hospital based, some data were missing. Although the study has been conducted over the period of three years, the seasonal variation couldn’t be elicited because of lack of data. Also, the domestic conflict could not be further elaborated because of inadequate data but it still gives a prominent and a larger view of how the family dispute is a major cause. The severity of the patient is only based on POP score, but different toxicological parameters could have contributed to the complications.

## Conclusion

Pesticides, commonly organophosphate compounds are used as the major means of self-poisoning. Our study showed that young adults and females are mostly involved in poisoning with the intent of suicide. Disputes with family members were found to be the main reason. Short-term suicidal impulses and the easy availability of pesticides also contributed to suicides. Limitations of a hospital-based study like ours can be overcome by a population-based study, which will provide true reflection about the problem of pesticide exposure and self-poisoning. Easy access to pesticides should be restricted with strong regulatory mechanisms and implementation of rules and regulations for pesticide handling. Early psychiatric consultation and identification of psychiatric disorders will help to reduce the incidence of self-harm. So, preventive strategies and mental health awareness programs should be conducted for high-risk populations.

## Data availability

### Underlying data

Figshare. Psychological and clinical-epidemiological profile of poisoning in Nepal: an institutional experience. DOI: https://doi.org/10.6084/m9.figshare.14776344.v1.

This project contains the following underlying data:

• Excel data OP.xlsx (de-identified data on psychological and clinical-epidemiological profile of patients visiting Scheer Memorial Adventist Hospital after poisoning presented from 1 ^st^ January 2018 to 31 ^st^ December 2020.)
• Final Data.dta (de-identified data on psychological and clinical-epidemiological profiles of patients visiting Scheer Memorial Adventist Hospital after poisoning presented from 1 ^st^ January 2018 to 31 ^st^ December 2020.)

And the following extended data:

• Supplementary analysis.docx (tables with details on occupation, poison taken, symptoms at presentation, complications and binary logistic regression analysis)
• Data collection sheets.docx (pre-specified data collection sheets which were used to collect data from individual patients file)

Data are available under the terms of the Creative Commons Zero “No rights reserved” data waiver (CC BY 4.0 Public domain dedication).

**Authors’ contributions**: AB, SC, and AT contributed to the concept, design, and interpretation of data. DBS guided, analyzed, and interpreted the finding. MK, BR, MK, KA, AJT, SP, KKS, PS, and SA contributed to the literature search, data extraction, and initial manuscript drafting. AB and DBS contributed in revising the manuscript for important intellectual content and approval of the final manuscript.

All authors were involved in drafting and revising the manuscript and approved the final version.
