# Supplementary material for: Psychological and clinical-epidemiological profile of poisoning in Nepal: an institutional experience
Source: F1000Res. 2021 Aug 31;10:556. Originally published 2021 Jul 12. [Version 2] doi: 10.12688/f1000research.54327.2 (PMC8329602; doi:10.12688/f1000research.54327.2)
Supplement: Peer review history

# Peer review: Reviewer response for version 1

**DOI:** 10.5256/f1000research.57804.r89443

**Published:** 2021-08-02

**Authors:** Nishita Pathak

**Affiliations:** 1 Civil Service Hospital, Kathmandu, Nepal

As a reviewer, I would like to highlight that the authors have nicely elaborated the psychological aspects of suicide including female dominance, domestic violence as the main cause, and organophosphorus poisoning being the most common. However, some points that I feel are lacking in this study are as follows: 

1. Whether the suicidal cause was impulsive or planned. These incidences seem to be important because planned suicides are more common in underlying mental health problems like major depressive disorder, old age, divorced, and homeless individuals ^1^. Impulsive suicides are more common in inter-personal conflicts which suggest a lack of coping.   
2. Domestic conflict if elaborated on with spouse or other family members would have been better. This point would strengthen the study as it would give some data as to the number of suicidal incidences when conflict would occur with spouse and other family members separately. ^2^

 '

Is the work clearly and accurately presented and does it cite the current literature?

Yes

If applicable, is the statistical analysis and its interpretation appropriate?

I cannot comment. A qualified statistician is required.

Are all the source data underlying the results available to ensure full reproducibility?

Partly

Is the study design appropriate and is the work technically sound?

Yes

Are the conclusions drawn adequately supported by the results?

Yes

Are sufficient details of methods and analysis provided to allow replication by others?

Yes

Reviewer Expertise:

Substance use disorder, Depression and anxiety ,

I confirm that I have read this submission and believe that I have an appropriate level of expertise to confirm that it is of an acceptable scientific standard.

## References

[1] : Differences between Impulsive and Non-Impulsive Suicide Attempts among Individuals Treated in Emergency Rooms of South Korea. *Psychiatry Investig* .2016;13(4) : 10.4306/pi.2016.13.4.389 389-96 10.4306/pi.2016.13.4.389 27482239PMC4965648

[2] : Family Conflict – The major underlying influence in suicide attempts in Northern Bihar, India. *Christian Journal for Global Health* .2015; 10.15566/cjgh.v2i1.52 23-34 10.15566/cjgh.v2i1.52

# Peer review: Reviewer response for version 1

**DOI:** 10.5256/f1000research.57804.r89442

**Published:** 2021-07-15

**Authors:** Swapnajeet Sahoo

**Affiliations:** 1 Assistant Professor, Department of Psychiatry, Institute of Medical Education and Research, Chandigarh, Haryana and Punjab, India

The materials and methods section should have a detailed summary of how the records are maintained in the hospital so that at any point of time these can be reverified. Like having unique registration number of the patient or a register where all poisoning cases are documented along with clinical variables and outcome parameters.

Is the work clearly and accurately presented and does it cite the current literature?

Yes

If applicable, is the statistical analysis and its interpretation appropriate?

Yes

Are all the source data underlying the results available to ensure full reproducibility?

Partly

Is the study design appropriate and is the work technically sound?

Yes

Are the conclusions drawn adequately supported by the results?

Yes

Are sufficient details of methods and analysis provided to allow replication by others?

Yes

Reviewer Expertise:

Consultation Liasion Psychiatry, Schizophrenia, Acute Psychosis, Psychosexual disorders

I confirm that I have read this submission and believe that I have an appropriate level of expertise to confirm that it is of an acceptable scientific standard.
